# Mammography to tomosynthesis: examining the differences between two-dimensional and segmented-three-dimensional visual search

**DOI:** 10.1186/s41235-018-0103-x

**Published:** 2018-06-14

**Authors:** Stephen H. Adamo, Justin M. Ericson, Joseph C. Nah, Rachel Brem, Stephen R. Mitroff

**Affiliations:** 1 Department of Psychology, The George Washington University, Washington, DC, USA; 2 Department of Radiology, The George Washington University, Washington, DC, USA

**Keywords:** Visual search, Mammography, Tomosynthesis, Segmented-three-dimensional search

## Abstract

**Background:**

Radiological techniques for breast cancer detection are undergoing a massive technological shift—moving from mammography, a process that takes a two-dimensional (2D) image of breast tissue, to tomosynthesis, a technique that creates a segmented-three-dimensional (3D) image. There are distinct benefits of tomosynthesis over mammography with radiologists having fewer false positives and more accurate detections; yet there is a significant and meaningful disadvantage with tomosynthesis in that it takes longer to evaluate each patient. This added time can dramatically impact workflow and have negative attentional and cognitive impacts on interpretation of medical images. To better understand the nature of segmented-3D visual search and the implications for radiology, the current study looked to establish a new testing platform that could reliably examine differences between 2D and segmented-3D search.

**Results:**

In Experiment 1, both professionals (radiology residents and certified radiologists) and non-professionals (undergraduate students) were found to have fewer false positives and were more accurate in segmented-3D displays, but at the cost of taking significantly longer in search. Experiment 2 tested a second group of non-professional participants, using a background that more closely resembled a mammogram, and replicated the results of Experiment 1—search was more accurate and there were fewer false alarms in segmented 3D displays but took more time.

**Conclusion:**

The results of Experiments 1 and 2 matched the performance patterns found in previous radiology studies and in the clinic, suggesting this novel experimental paradigm potentially provides a flexible and cost-effective tool that can be utilized with non-professional populations to inform relevant visual search performance. From an academic perspective, this paradigm holds promise for examining the nature of segmented-3D visual search.

## Significance 

This study is the first step in establishing a new paradigm to examine segmented-three-dimensional (3D) visual search that can be used with professional and non-professional searchers, which has theoretical and real-world implications. Theoretically, while there is a long history of studying the nature of visual search in cognitive psychology, visual search in a segmented-3D environment has been relatively unexplored. This new paradigm can add to existing classic theories with the potential to generate novel ones. Practically, it is possible to learn about breast cancer detection by studying how non-professionals search for targets in a two-dimensional (2D) environment compared to a segmented-3D environment. The findings from this study replicated the results typically found in radiology when comparing breast cancer detection in mammography (a 2D radiograph of breast tissue) to tomosynthesis (a segmented-3D tomogram of breast tissue)—search in 2D is less accurate and quicker. Importantly, the results were found with both professional and non-professional populations, suggesting this may be a general search attribute that can be observed in different populations. Since professionals are difficult to recruit as participants, it is potentially quite powerful that this paradigm can be run with non-professional participants knowing they demonstrate similar patterns in search performance found within radiology. Likewise, it is promising that we replicated the pattern of results found with tomosynthesis radiographic images with the use of simple stimuli given that there is more experimental control with simple stimuli and they can be shown to non-professionals. The primary significance of this project is that it takes the first steps in establishing a new cognitive psychology paradigm that can inform the growing field of tomosynthesis for breast cancer detection.

## Background

For decades, mammography has been the primary tool of choice for radiologists who are tasked with detecting breast cancer (Bleyer & Welch, 2012). Mammography is the process of creating a single 2D image that represents an entire breast (a mammogram) and then examining that image for signs of cancer. Despite extensive training and often years (or decades) of experience, radiologists are not perfect and will miss present abnormalities in a mammogram (Rosenberg et al., 1998).

Radiologists can miss a cancer for a variety of reasons and the focus on the current study is on one particular cause—the limits of human cognitive processing. This is a well-known contributor in radiological misses, with several robust examples. For example, radiological misses are more likely to occur when targets are rarely present (Evans, Birdwell, & Wolfe, 2013) and after another abnormality has already been found in the same read (e.g. Berbaum et al., 1991). These two specific negative impacts on radiological success (low target prevalence and “satisfaction of search,” respectively) have been studied by radiologists for decades, with several insights gained. Interestingly, these two specific cases have also been examined via basic psychology studies that have used simplified displays and non-professional searchers (e.g. undergraduate participants in a psychology study). Such psychology studies have been able to provide radiologically relevant conclusions about the limitations of cognitive processing related to target prevalence (e.g. Van Wert, Horowitz, & Wolfe, 2009; Wolfe, Horowitz, & Kenner, 2005; however, see Fleck & Mitroff, 2007) and target number (e.g. Adamo, Cain, & Mitroff, 2013, 2017; Fleck, Samei, & Mitroff, 2010).

To overcome the limits of human cognitive processing, radiology has often looked to technological aids. The goal is to use advances in technology to counter inevitable cognitive failures. For example, computer-aided detection (CAD), a recognition software that highlights potential cancers in a radiograph, can be used by radiologists to potentially aid them in cancer detection (e.g. Lehman et al., 2015). However, while CAD can improve search performance (e.g. Brem, Hoffmeister, Zisman, DeSimio, & Rogers, 2005; Zheng et al., 2001), it is not infallible and has been shown to lead to no improvement in accuracy (e.g. Lehman et al., 2015) and even more misses under conditions when multiple abnormalities are present (e.g. Berbaum, Caldwell, Schartz, Thompson, & Franken Jr., 2007).

The radiological field of breast imaging is currently undergoing a new technological shift to improve breast cancer detection by changing how radiologists view medical images. Specifically, imaging is moving away from mammography, a 2D imaging technique, to tomosynthesis, a segmented-3D imaging technique. Unlike mammography, where the volume of the breast is compressed into one 2D image, tomosynthesis is the process of dividing the volume of the breast into many segmented images (i.e. slices) to create a segmented-3D display. With tomosynthesis, radiologists have the ability to search in depth by moving from slice-to-slice allowing them to better distinguish signs of cancer from normal breast tissue. Tomosynthesis has been a success to date; with tomosynthesis, radiologists tend to make fewer false positives/false alarms (e.g. incorrectly indicating a benign mass as malignant; Durand et al., 2015; Friedewald et al., 2014; Skaane et al., 2013) and detect cancer more often (Ciatto et al., 2013). However, this improvement comes at a cost as it takes radiologists significantly longer, even up to double the amount of time, to evaluate a patient with a combined tomosynthesis and mammography read compared to mammography alone (Bernardi et al., 2012; Dang, Freer, Humphrey, Halpern, & Rafferty, 2014; Michell et al., 2012; Zuley et al., 2010). The increase in evaluation time is not a subtle point as this has put enormous stress on the workload of radiologists. In general, overwork can lead to several negative outcomes, including more missed cancers (e.g. Krupinski, Berbaum, Caldwell, Schartz, & Kramer, 2012) and legal concerns (e.g., Berlin, 2000).

Beyond the clinical effects of tomosynthesis, there is little work on the nature of searching in a segmented-3D environment. While there is a growing literature of visual search in 3D environments created through stereoscopic techniques (i.e. presenting different views to the right and left eye to induce a 3D effect; e.g. Finlayson, Remington, Retell, & Grove, 2013; McIntire, Havig, & Geiselman, 2014), only recently has there been research on searching through environments with successive slices that allow the viewer to move in and out of the depth plane (e.g. Drew et al., 2013; Wen et al., 2016). For example, one recent study (Wen et al., 2016) found that different search styles within a segmented-3D environment changes what is more salient to an observer. Specifically, when observers “drilled” (i.e. staring at a region within a segmented-3D environment and rapidly scroll from slice to slice through the depth plane) they were drawn toward 3D dynamic motion saliency, and when observers “scanned” (i.e. searching over a large area of a given slice before moving to the next slice) they were drawn toward 2D saliency. With the ability to utilize different search styles and the ability to search in depth, this raises questions as to what the cognitive processes underlying segmented-3D search are and how they compare to that of 2D search.

The current study used a simplified cognitive psychology paradigm to examine 2D and segmented-3D search in both professional (radiology residents and certified radiologists) and non-professionals (undergraduate students). Beyond the theoretical reasons for comparing search performance in a 2D and segmented-3D environment, the main motivation for conducting this experiment was to explore whether the results would yield similar findings to that of radiologists when using mammography and tomosynthesis in practice. If this novel lab-based task replicated the pattern of results found within radiology (i.e. segmented-3D search/tomosynthesis revealing decreased false alarms, increased hits, and increased search times), the control and flexibility of this program could be used to better understand what underlies the differences in search performance. Another key motivation for this experiment was to compare performance between professionals and non-professionals. If non-professionals performed similarly to professionals, future research could explore segmented-3D search with non-professionals and gain insight as to how professionals would perform with tomosynthesis. Since professionals are difficult to access as participants in research studies (due to their time constraints), and there is less experimental control when using medical images, testing non-professionals in a laboratory based, segmented-3D search would be a faster, easier, and more flexible alternative. This experimental path mirrors prior efforts from our research team that created a simplified paradigm that could be used with non-professionals (Fleck et al., 2010) to potentially inform radiological questions (e.g. Adamo, Cain, & Mitroff, 2015; Cain, Dunsmoor, LaBar, & Mitroff, 2011; Cain & Mitroff, 2013).

To preview the results, professionals and non-professionals replicated the pattern of results found within radiological practice—there were fewer false alarms, better accuracy, and longer response times in segmented-3D search compared to 2D search. Despite many differences between the two participant populations (e.g. search experience, age), there were no significant differences in search performance.

## Experiment 1

### Methods

#### Participants

##### Professionals

A total of 30 participants composed of radiology residents and certified radiologists were recruited from the Radiological Society of North America conference in Chicago, Illinois between 27 November 27 and 2 December 2016. This sample size was determined by how many professionals could be recruited at the conference. The participants had no restriction on their specialty (e.g. breast, thoracic, general) and were entered in a drawing for a chance to win a GoPro Hero 4 for their participation. Three participants were not included in the final analysis: two were removed due to experimental error during data collection and one for quitting the experiment early, leaving a total of 27 participants (14 radiology residents and 13 certified radiologists). The 27 participants’ age range was 24–65 years (radiology residents: age range = 24–39 years, M = 30.69, SD = 3.88; certified radiologists: age range = 33–65 years, M = 46.92, SD = 10.66) and there were 11 women and 16 men. The average number of cases evaluated per week was in the range of 50–600 (radiology residents: range = 50–300, M = 150.91, SD = 93.96; certified radiologists: range = 100–600, M = 340.00, SD = 207.67). There were overall no differences in search performance between radiology residents and certified radiologist (see Appendix 1), so they were treated as a single population group (professionals) when compared to undergraduate students.

##### Non-professionals

A total of 31 undergraduate students (non-professionals) were recruited from The George Washington University to approximately match the number of recruited professionals. They had no radiology experience and received course credit in exchange for participation. There were 20 women and 11 men with an age range of 18–23 years (mean age = 18.8 years; SD = 1.0).

#### General procedures

Participants sat approximately 45 cm (with no head restraint) from the center of a 13-in. MacBook Pro laptop computer. Stimulus displays were presented using Matlab software (The MathWorks, Natick, MA, USA) and Psychophysics Toolbox 3.0.12 (Brainard, 1997; Pelli, 1997) at a resolution of 800 × 600 pixels. The search displays were constructed in 3D space, filling in a cube array that was 600 × 600 × 600 voxels.^1^ This cube was then trimmed into a 600-voxel diameter sphere. To generate a cloudy background akin to a mammogram, 250 ellipsoids (i.e. clouds) in the range of 50–350 voxels were created and randomly placed in the sphere. Afterwards, a 3-voxel Gaussian filter was applied twice to smooth the image.

**Fig. 1:**
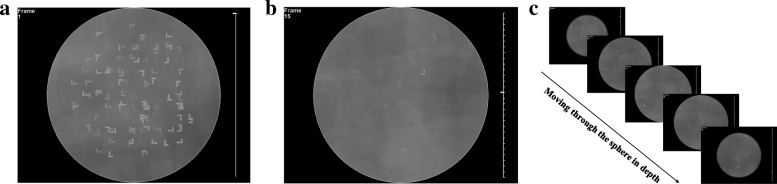
Experiment 1’s 2D- and segmented-3D-search displays. **a** Sample stimuli of a 2D-search display where all search items appear in a single “flat” plane. **b** Sample stimuli of a slice in the segmented-3D-search display. Search items were randomly assigned to each slice, and unlike in the 2D display, did not all appear on the same depth plane. **c** *Illustration* of how participants could move from slice to slice to search in depth in the segmented-3D displays

Target-present displays contained one T-shaped target and 99 L-shaped distractors and target-absent displays contained 100 L-shaped distractors. Each item (target T or distractor L) could be rotated 0, 90°, 180°, or 270° along the y-axis (see Fig. 1) and was 15 × 7 × 17 voxels (0.89° × 0.64° × 1.3°) for the X, Y, and Z coordinates, respectively. The T-shaped targets were constructed with two perfectly aligned perpendicular cross bars and the two cross bars were offset by 3 voxels (0.27°) to form non-perfect, L-shaped distractors. Both the targets and distractors had a 3-voxel gap between the cross bars. Colors of the search items were randomly selected within a gray-scale range of 47–63% white. The search items were randomly placed within a 15 × 15 × 15 location matrix for the 3D displays. The matrix was transcribed into the sphere and any cells that overlapped the perimeter of the sphere were removed, so no target or distractor could appear outside the display area. The search items were then jittered by 0–16 pixels along the x- and y-axes and by 0–32 pixels in the z-axis for the 3D displays.

Spheres were either compressed into a single “flat” plane for the 2D-search displays (see Fig. 1a) or were divided and compressed into 30 different slices for the 3D-search displays (see Fig. 1b and c). When compressing the sphere array for the 2D-search displays the average pixel color (for both items and the background) for a given x- and y-coordinate voxel was averaged between the middle three slices to create a single, 2D search plane. For the 3D search displays, each slice was similarly computed by taking the average colors of the pixels across every 20 voxels’ z-coordinate. This process created 30 slices per display, effectively making it a “segmented-3D” display wherein search items could be contained within one slice or across two slices.^2^ This process caused a high probability that the search items would overlap in the 2D displays and a low-probability of overlap on each slice in the segmented-3D displays (see Fig. 1).

A tick bar appeared on the right side of the displays (see Fig. 1), with each tick representing one slice in the depth plane. A marker moved as participants traversed from slice to slice and indicated which slice the participant was currently on when searching through the segmented-3D displays. The slice number was also presented at the top left of the displays (see Fig. 1). For the 2D-search displays, there was a single tick mark and the number in the top left corner displayed the number “1.”

There were four practice trials and 24 experimental trials. The practice trials were separated into two blocks (2D, segmented-3D) and the experimental trials were separated into four blocks (two 2D blocks and two segmented-3D blocks) with an equal number of trials per block. Block order was randomized for the practice. Block order was also randomized for the first two experimental blocks and then again for the last two experimental blocks. The trials in the last two experimental blocks were repeats of the trials from the first two experimental blocks, but in the opposite display type (e.g. the x and y coordinates for targets and distractors in the segmented-3D trials from the first half of the experiment were repeated for the 2D trials in the second half of the experiment). There was an equal number of target-present and target-absent trials with a randomized and equal distribution of trials per block.

For the segmented-3D displays, participants used a mouse wheel to scroll from slice to slice and for the 2D displays the mouse wheel was not used. Participants were instructed to indicate the target location via a mouse click and press the spacebar if they believed no target was present. The trial ended once either a mouse click was made or spacebar was pressed. If participants reached a 60-s time limit without making a response, the trial ended and was considered a “timeout.” The tick bar turned yellow after 50 s and then red after 55 s to inform participants that the trial was about to end. The next trial loaded during the inter-trial interval and started immediately once loaded. Similarly, after each block, the next block automatically began. The experiment took approximately 30 min in total.

#### Planned analyses

The primary goal of Experiment 1 was to explore whether the results would yield similar findings to that of radiologists when using tomosynthesis and mammography. When comparing tomosynthesis to mammography, there are fewer false alarms (e.g. Skaane et al., 2013), improved cancer detection (Ciatto et al., 2013), and a significantly longer time spent evaluating a patient’s case (e.g. Bernardi et al., 2012). As such, the three respective key measures of interest for Experiment 1 were false alarm rate, hit rate, and target-absent response time. Target-absent response time was assessed because it represents how long a participant will spend searching before deciding to quit (Chun & Wolfe, 1996). There are additional measures of interest that are standard dependent variables in visual search experiments (e.g. timeout rate, target-present response time) but they were not primarily relevant for the current study and can be found in Appendix 2.

Note that trials where participants timed out were excluded for the hit rate analysis and trials where participants timed out or false alarmed were excluded from the target-absent response time analysis. Also, while the goal of repeating trials from experimental blocks 1 and 2 in blocks 3 and 4 in the other display type was to explore differences between the repeated and initial trials, there were no meaningful differences based on repetition. Specifically, there was no significant difference in search performance between the 2D trials within the first half of the experiment (where the x and y coordinates of a specific 2D trial were seen for the first time) and the 2D trials in the second half of the experiment (where the x and y coordinates previously seen within the segmented-3D display of the first half of the experiment were repeated in the 2D displays). Likewise, there were no significant differences in performance for the segmented-3D trials from the first half of the experiment and those from the second half. As such, the repetition aspect of the study design will not be discussed further.

### Results

For each of the three primary dependent variables of interest (false alarm rate, hit rate, and target-absent response time), a 2 × 2 analysis of variance (ANOVA) was run with search display as a within-subjects factor (2D, 3D) and profession as a between-subjects factor (professionals, non-professionals). For brevity, we refer to the segmented-3D displays in the results section as just “3D.” Below is an overview of the results and the details are in Table 1.

#### False alarm rate

**Fig. 2:**
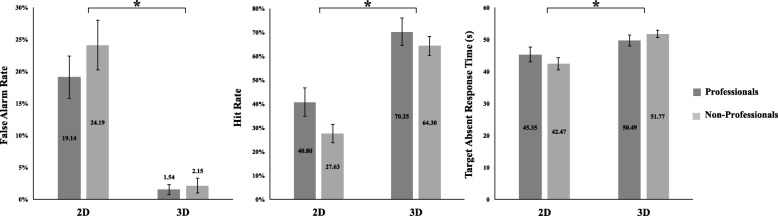
Key measures for professionals and non-professionals in 2D and segmented-3D visual search in Experiment 1. The *asterisks* indicate a main effect of display type: false alarm rates were lower, hit rates were higher, and target-absent response times were longer for segmented-3D displays compared to 2D displays. *Error bars* represent the standard error of the mean

On average, the false alarm rate was 10.34% for professionals (2D: 19.94%; 3D: 1.49%) and 13.17% for non-professionals (2D: 24.19%; 3D: 2.15%). There was a statistically significant main effect of display type (*F*(1,56) = 71.48, *p* < 0.001, η_p_^2^ = 0.56, BF_10_ = 4.24 × 10^10^)^3^ with greater false alarms on 2D-search trials compared to 3D trials (See Fig. 2). There was no significant main effect of profession (*F*(1,56) = 0.89, *p* = 0.35, η_p_^2^ = 0.02, BF_10_ = 0.29) and no significant interaction between display type and profession (*F*(1,56) = 0.90, *p* = 0.35, η_p_^2^ = 0.02, BF_10_ = 0.40).

#### Hit rate

On average, the hit rate was 55.52% for professionals (2D: 40.80%; 3D: 70.25%) and 45.97% for non-professionals (2D: 27.63%; 3D: 64.30%). Data from one professional were not included in the final analysis as that individual timed out on all 3D trials. There was a statistically significant main effect of display type (*F*(1,56) = 59.73, *p* < 0.001, η_p_^2^ = 0.51, BF_10_ = 1.23 × 10^9^) with a lower hit rate on 2D search trials compared to 3D trials (see Fig. 2). There was no significant main effect of profession (*F*(1,56) = 3.11, *p* = 0.08, η_p_^2^ = 0.05, BF_10_ = 0.60), and no significant interaction between display type and profession (*F*(1,56) = 0.71, *p* = 0.40, η_p_^2^ = 0.01, BF_10_ = 0.41).

#### Target-absent response times

On average, the target-absent response time was 47.92 s for professionals (2D: 45.35 s; 3D: 50.49 s) and 47.12 s for non-professionals (2D: 42.47 s; 3D: 51.77 s). Data from two professionals were not included in the final analysis because they timed out or false alarmed on either all 2D or 3D trials. There was a statistically significant main effect of display type (*F*(1,54) = 43.12, *p* < 0.001, η_p_^2^ = 0.43, BF_10_ = 6.78 × 10^6^) with a shorter target-absent response time on 2D trials compared to 3D trials (see Fig. 2). There was no significant main effect of profession (*F*(1,54) = 0.13, *p* = 0.73, η_p_^2^ = 0.002, BF_10_ = 0.29) and no significant interaction between display type and profession (*F*(1,54) = 3.57, *p* = 0.06, η_p_^2^ = 0.002, BF_10_ = 1.19).

**Table 1:** Summary of Experiment 1 results for professionals and non-professionals

Key measures	Radiologists		Non-professionals		Display type (2D vs 3D)	Profession (Professionals vs. non-professionals)	Interaction
	2D	3D	2D	3D			
False alarm rate (%)	19.14	1.54	24.19	2.15	***p*** **< 0.001**, η_p_^2^ = 0.56, *BF*_*10*_ *= 4.24 × 10*^*10*^	*p* = 0.35, η_p_^2^ = 0.02, *BF*_*10*_ *= 0.29*	*p* = 0.35, η_p_^2^ = 0.02, *BF*_*10*_ *= 0.40*
Hit rate (%)	40.80	70.25	27.63	64.30	***p*** **< 0.001**, η_p_^2^ = 0.51, *BF*_*10*_ *= 1.23 × 10*^*9*^	*p* = 0.08, η_p_^2^ = 0.05, *BF*_*10*_ *= 0.60*	*p* = 0.40, η_p_^2^ = 0.01, *BF*_*10*_ *= 0.41*
Target-absent response time (s)	45.35	50.49	42.47	51.77	***p*** **< 0.001**, η_p_^2^ = 0.43, *BF*_*10*_ *= 6.78 × 10*^*6*^	*p* = 0.73, η_p_^2^ = 0.00, *BF*_*10*_ *= 0.29*	*p* = 0.06, η_p_^2^ = 0.04, *BF*_*10*_ *= 1.19*

For the key measures, there was a lower false alarm rate, a higher hit rate, and a longer average response time on target-absent trials for segmented-3D displays compared to 2D displays. *P* values < 0.05 are indicated in bold and the Bayes factors are indicated in italics

### Discussion

Search performance on 2D displays compared to segmented-3D displays mirrored how radiologists typically perform when comparing mammography to tomosynthesis: false alarm rates (false positives) were higher, hit rates (true positives) were lower, and target-absent response times were shorter for 2D displays compared to segmented-3D displays. The Bayes factor for the main effect of displays provided “decisive” evidence in a favor of the alternative hypothesis suggesting that there was a difference between display types (Wetzels et al., 2011). Additionally, while the interaction effect for target absent RT was marginally significant (*p* = 0.06), the Bayes factor provided “anecdotal evidence” in support of the alternative hypothesis. This appears to be driven by a larger difference between 2D and segmented-3D display types for non-professionals compared to professionals. The current results were encouraging given that the findings replicated the results typically found within radiology. However, one potential limitation of this study was that the background in the search displays was not realistic in comparison to actual radiographs. Experiment 2 accounted for this by using a more realistic background that has been shown to more closely resemble an actual mammogram (Castella et al., 2008).

## Experiment 2

To ensure that the results from Experiment 1 could be replicated and were not driven by the background of the stimulus displays, the displays of Experiment 2 consisted of a more realistic mammography background (Castella et al., 2008). To preview the results, in Experiment 2 non-professionals replicated the pattern of results found within radiological practice and Experiment 1—there were fewer false alarms, better accuracy, and longer response times in segmented-3D search compared to 2D search.

### Methods

#### Participants

Thirty-one undergraduate students were recruited from The George Washington University. One participant reported previous radiological experience; therefore, his/her data were removed from the analyses. In addition, one participant’s data were removed as she/he timed out on too many trials (more than 3 standard deviations over the mean). The remaining 29 participants comprised 22 women and seven men with an age range of 18–23 years (mean age = 19.7, SD = 1.08). They had no previous radiology experience and received course credit in exchange for participation.

#### General procedures

**Fig. 3:**
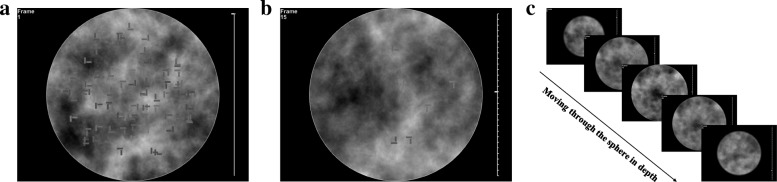
Experiment 2’s 2D and segmented-3D search displays. **a** Sample stimuli of a 2D-search display where all search items appear in a single “flat” plane. **b** Sample stimuli of a slice in the segmented-3D-search display. Search items were randomly assigned to each slice, and unlike in the 2D display, did not all appear on the same depth plane. **c** *Illustration* of how participants could move from slice-to-slice to search in depth in the segmented-3D displays

The methods were the same as Experiment 1 except for the following three changes. First, the display backgrounds (see Fig. 3) were created by Castella et al. (2008) to encourage “drilling” behavior while searching the segmented-3D displays as the search items would blend better with the background (Drew et al., 2013). Castella et al. (2008) generated a number of possible backgrounds and found that one, which was called “doubiso,” best approximated mammograms. As such, the doubiso synthetic mammographic texture was used here. To incorporate the doubiso image into the 3D sphere for our segmented-3D displays, each trial contained three randomly generated doubiso images that were placed at the start, middle, and end of the sphere along the depth plane. The intermediate points were averaged between these three images to create a continuous space. The 30 slices were created as described for Experiment 1. For the 2D displays, only one doubiso synthetic texture was used as the search display. Second, there were 80 items per search (as opposed to 100), each with a dimension of 20 × 7 × 22 voxels (1.19° × 0.64° × 1.3°), and a color range of 28–47% white. Third, there were 48 experimental trials (12 trials per block) with four practice trials and observers had up to 1 h to complete the experiment. Doubling the number of trials from Experiment 1, allowed the opportunity to explore whether there were any differences in sensitivity (A’) and bias (B”) between the two display types.^4^

### Results

T-tests were conducted to compare the false alarm rate, hit rate, and target-absent response times on 2D vs segmented-3D search displays. Similar to Experiment 1, the timeout rate, the correct rejection rate, and the target-present response time were also assessed and are reported in Appendix 2. For brevity, we refer to the segmented-3D displays in the results section as just “3D.”

Non-professionals in Experiment 2 had an average false alarm rate of 19.18%. There was a statistically significant difference between display types (*t*(28) = 6.19, *p* < 0.001, BF_10_ = 1.62 × 10^4^) with greater false alarms on 2D trials (M = 27.59%) compared to 3D trials (M = 10.78%). The average hit rate was 42.26%. There was a statistically significant difference between display types (*t*(28) = 5.93, *p* < 0.001, BF_10_ = 8.46 × 10^3^) with a lower hit rate on 2D trials (M = 29.34%) compared to 3D trials (M = 55.28%). The average target-absent response time was 41.87 s. There was a statistically significant difference between display types (*t*(28) = 7.67, *p* < 0.001, BF_10_ = 5.84 × 10^5^) with a shorter target-absent response time on 2D trials (M = 35.06 s) compared to 3D trials (M = 47.10s).

**Fig. 4:**
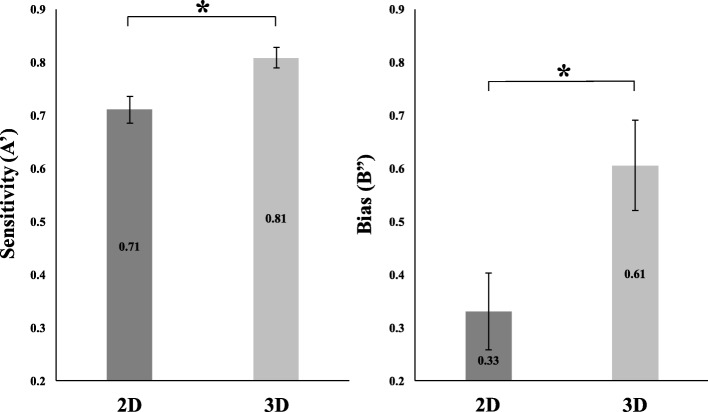
Sensitivity (A’) and bias (B”) measures for 2D- and segmented-3D-search displays in Experiment 2. The *asterisks* indicate a significant difference between A’ and B” between 2D and segmented-3D displays with a greater A’ and B” in segmented-3D displays

Observers were above chance for sensitivity to targets (i.e. more likely to identify a search item as a target) in both 2D (A’ = 0.71; *t*(28) = 8.44, *p* < 0.001, BF_10_ = 3.46 × 10^6^) and segmented-3D displays (A’ = 0.81; *t*(28) = 15.82, *p* < 0.001, BF_10_ = 3.44 × 10^12^). Furthermore, they were more sensitive in segmented-3D displays compared to 2D displays (*t*(28) = 2.91, *p* = 0.007, BF_10_ = 6.15). Observers were conservative in their bias (i.e. more likely to identify a search item as a target) in both 2D (B” = 0.33; *t*(28) = 4.61, *p* < 0.001, BF_10_ = 3.15 × 10^2^) and segmented-3D displays (B” = 0.61, *t*(28) = 7.13, *p* < 0.001, BF_10_ = 1.59 × 10^5^). Additionally, they were more conservative in their bias for segmented-3D displays compared to 2D displays (*t*(28) = 3.24, *p* = 0.003, BF_10_ = 12.63) (see Fig. 4).

### Discussion

When using a more realistic mammographic background in 2D and segmented-3D- search displays, the results replicated the typical findings from radiology (when comparing mammography to tomosynthesis) and the key findings from Experiment 1; the false alarm rates were lower, the hit rates were higher, and the response times for target-absent trials were longer for segmented-3D displays compared to 2D. This replication reinforces the results from Experiment 1 and further suggests that the paradigm assessed here can potentially be used to examine the differences between mammography and tomosynthesis. Furthermore, with an increase in trials from Experiment 1, we could explore whether there was a difference in sensitivity and bias between the 2D and segmented-3D displays. Observers were more sensitive to targets and more conservative in their bias in the segmented-3D displays compared to the 2D displays. These analyses provided additional evidence for the benefits of searching through segmented-3D displays compared to 2D displays.

## General discussion

As radiology practices move from relying on mammography to tomosynthesis for breast cancer detection, it is critical to understand the potential positives and negatives of the relatively new technology of tomosynthesis. Academic radiology has begun the process of examining tomosynthesis (e.g. Skaane et al., 2013) but many questions remain. The goal of the current study was to explore whether a basic cognitive psychology paradigm, utilizing non-professional participants, could be leveraged to further inform the use of tomosynthesis. The initial step in this process was creating a 2D and segmented-3D visual search paradigm and explore whether search performance mimicked the results when comparing mammography to tomosynthesis.

In Experiment 1, both professionals (radiology residents and certified radiologists) and non-professionals (undergraduate students) searched through 2D and segmented-3D displays with a cloudy gray background. The results replicated the pattern of findings from the radiological literature (e.g. Bernardi et al., 2012; Ciatto et al., 2013; Skaane et al., 2013)—there was a lower false alarm rate, a higher hit rate, and more time was spent determining that a target was absent on segmented-3D displays compared to 2D displays. In Experiment 2, a second group of non-professionals conducted this search paradigm with a more realistic mammography-like background. The results again replicated the pattern of findings from the radiological literature.

While there was no main effect of search performance between professionals and non-professional, this does not necessarily indicate there are no differences in search ability. It is notoriously difficult to reason from a null result and there could be any number of additional factors at play. For example, it could be that radiologists did have superior search abilities but demonstrated no difference in search performance as a result of the 60 s time restriction and the one target (at most) per search display. While radiologists may have to inspect a set number of medical images per day, there is no time limit to how long radiologists can spend per image or how many abnormalities may be present in a given image. If given more time and/or more targets per search display, it may have been possible to reveal differences between the population groups.

Another possible reason there were no significant differences in search performance between professionals and non-professional was that any potential search advantage that professionals had due to experience could have been offset by their age. Professionals on average were older than the non-professional participants (see “Methods” for Experiment 1) and it is known that older adults typically show a cognitive decline in attention and memory (e.g. Fortenbaugh et al., 2015). That is, it could be that our two participant groups look similar in raw performance and in terms of statistical comparisons, but that they might differ in underlying abilities. However, a similar study comparing aviation security officers and undergraduates found that aviation security officers are better than undergraduates at a simplified visual search task (Biggs, Cain, Clark, Darling, & Mitroff, 2013). Moreover, there is additional insight from a study that compared orthodontists (who specialize in facial symmetry) to undergraduate participants (Jackson, Clark, & Mitroff, 2013). The orthodontists (who were, on average, older than the undergraduates) were more accurate on a facial symmetry judgement task but not on a non-facial symmetry judgement task. This suggests that age is not the sole factor in determining performance differences. While a potential age-related effect is just one hypothesized difference, the key point is that the null result for population differences should be evaluated in the context that there could be additional differences between populations that affect performance that could not be observed due to the given parameters of this study.

A key goal of the current project was to generate a simulated paradigm that could be used to assess cognitive differences between mammography and tomosynthesis. As such, it is important to compare performance found here to what is typically found in the clinic. One aspect that stands out is that when comparing false alarm rates (i.e. false positives), hit rates, and response times differences between mammography and tomosynthesis from the radiological literature (e.g. Bernardi et al., 2012; Ciatto et al., 2013; Skaane et al., 2013) to the results reported here, our results yielded a smaller percent difference between 2D and segmented-3D searches. For example, it took radiologists approximately 100% longer when searching with tomosynthesis compared to mammography alone (Bernardi et al., 2012). In our study it took participants 10–20% longer in Experiment 1 and about 35% longer in Experiment 2 to terminate search in segmented-3D searches compared to 2D searches. The larger difference within radiology could be driven by the inherent variability in difficulty when searching actual medical images. The displays used in this study were relatively the same difficulty; there was always one target, the same amount of items per search, and the same number of slices per search for the segmented-3D search displays. In medical images, difficulty may be potentially affected by the density of breast tissue, the number of abnormalities in a display, and the number of slices per tomogram.

In future studies, potential limitations of this study, such as the density of the background, the number of targets, and the number of slices per search display (for segmented-3D searches), can be modulated from trial to trial to observe whether they can influence the differences between the displays used here and actual medical images. In addition to these limitations, another potential limitation is how observers searched within our segmented-3D displays. Previous studies utilizing eye tracking observed that radiologists both “drilled” (i.e. staring at a region within a segmented-3D environment and rapidly scrolling from slice to slice) and “scanned” (i.e. searching over a large area of a given slice before moving to the next slice) volumetric images such as the segmented-3D used in this study (Drew et al., 2013). In Experiment 1, radiologists answered follow-up questions and self-reported utilizing both drilling and scanning techniques. However, 85% of radiologists that chose to respond to these questions reported that they exclusively scanned the segmented-3D images suggesting that these displays could have been searched differently than the volumetric displays used within radiology. The background of Experiment 2 was designed to encourage more drilling behavior (as items tended to blend better with the background), but this can be empirically examined in future studies (e.g. by monitor eye movements to assess whether observers are “drilling” and/or “scanning”).

Since this is one of the few studies to use a cognitive psychology paradigm to explore search performance on segmented-3D displays (e.g. Wen et al., 2016), it is important to consider how search performance is affected when searching in other 3D environments (i.e. non-segmented 3D displays). The main difference between the segmented-3D displays used here and other non-segmented-3D displays is that the illusion of depth for segmented-3D displays is created by having search items persist across continuous slices. Non-segmented-3D displays create the illusion of depth by presenting different views to the right and left eye (either via a stereoscope or with virtual reality goggles). Interestingly, the current finding of an improvement in target detection for the segmented-3D displays aligns with typical results found when comparing non-segmented-3D displays to 2D displays (e.g. Finlayson et al., 2013; McKee, Watamaniuk, Harris, Smallman, & Taylor, 1997). Much is left to explore, but the data collectively fit with a hypothesized improvement in 3D search via a reduction in local clutter (noise/distractors within the vicinity of a target; see Figs. 1b and 3b). A decrease in the amount of local clutter around a target has been shown to improve search performance in both single-target and multiple-target searches (Adamo et al., 2015; Asher, Tolhurst, Troscianko, & Gilchrist, 2013), so this could be a viable mechanism at play here. Future research could vary local clutter in 2D, segmented-3D, and non-segmented 3D search displays to help determine whether this is one of the reasons for an improvement in target detection when searching in 3D.

Beyond the theoretical implications this study has for understanding the nature of 3D search, practically this study is the first step in studying the differences between tomosynthesis and mammography without actual medical images and with a non-professional population. Since the results replicated the findings from the radiological literature when comparing mammography to tomosynthesis, this suggests that artificially created 2D and segmented-3D-search displays can be beneficial in understanding the differences between mammography and tomosynthesis. Furthermore, since there was no difference between professionals and non-professionals from Experiment 1, the results from non-professionals on segmented-3D search displays may provide insight as to how professionals would perform when searching with tomosynthesis. This is quite advantageous from a research viewpoint because radiologists can be difficult to recruit as participants (due to their time constraints) and there is less control over, and flexibility with, medical images as stimuli. Testing non-professionals in a segmented-3D-search paradigm could provide a faster, easier, and cheaper alternative. Future research can take advantage of using 2D and segmented-3D-search displays to explore other critical factors that have been shown to affect search performance (e.g. clutter and low-target prevalence).

## Conclusion

The current study investigated whether a cognitive psychology paradigm could be used to inform the use of tomosynthesis in breast cancer detection. We created 2D and segmented-3D visual search paradigms to explore whether the search performance of both professionals and non-professionals mimicked the patterns of search performance observed when comparing mammography to tomosynthesis in the clinic. The results matched the performance patterns from the radiological literature—a lower false alarm rate, a higher hit rate, and more time spent searching on segmented-3D displays compared to 2D displays (e.g., Bernardi et al., 2012; Ciatto et al., 2013; Skaane et al., 2013). These findings suggest that artificially created 2D and segmented-3D-search displays can potentially be a valid tool to help elucidate the difference between mammography and tomosynthesis.

## Appendix 1

### Comparing radiology residents to certified radiologists

**Table 2:** Summary of results for the key and other measures from radiology residents and certified radiologists for Experiment 1

	Radiology residents		Certified radiologists		Display type (2D vs 3D)	Profession (Radiological residents vs certified radiologists)	Interaction
	2D	3D	2D	3D			
Key measures							
False alarm rate (%)	14.88	2.38	23.71	0.64	*p* < 0.001, η_p_^2^ = 0.57, BF_10_ = 3.34 × 10^4^	*p* = 0.33, η_p_^2^ = 0.04, BF_10_ = .39	*p* = 0.10, η_p_^2^ = 0.10, BF_10_ = 1.16
Hit rate (%)	47.26	79.76	33.85	60.00	*p < 0.001*, η_p_^2^ = 0.39, BF_10_ = 1.96 × 10^2^	*p* = 0.07, η_p_^2^ = 0.12, BF_10_ = 0.87	*p* = 0.67, η_p_^2^ = 0.01, BF_10_ = 0.39
Target- absent response time (s)	47.07	51.25	43.48	50.07	*p* < 0.01, η_p_^2^ = 0.36, BF_10_ = 19.67	*p* = 0.56, η_p_^2^ = 0.02, BF_10_ = 0.52	p = 0.35, η_p_^2^ = 0.04, BF_10_ = 0.49
Other measures							
Timeout rate (%)	17.26	10.11	6.41	11.54	*p* = 0.70, η_p_^2^ = 0.01, BF_10_ = .29	*p* = 0.44, η_p_^2^ = 0.02, BF_10_ = 0.56	*p* = 0.03, η_p_^2^ = 0.18, BF_10_ = 2.58
Correct rejection rate (%)	71.55	85.95	70.00	98.61	*p* < 0.01, η_p_^2^ = 0.27, BF_10_ = 14.17	*p* = 0.27, η_p_^2^ = 0.06, BF_10_ = 0.50	*p* = 0.51, η_p_^2^ = 0.02, BF_10_ = 0.40
Target- present response time (s)	20.46	26.53	18.16	27.39 s	*p* < 0.01, η_p_^2^ = 0.38, BF_10_ = 29.46	*p* = 0.83, η_p_^2^ = 0.00, BF_10_ = 0.39	*p* = 0.47, η_p_^2^ = 0.03, BF_10_ = 0.45

Trials where participants timed out were excluded for the hit rate and correct rejection analyses, trials where participants timed out or false alarmed were excluded from the target-absent response time analysis, and trials where a participant missed a target, timed out, or false alarmed were excluded from the target-present response time analysis

The above table provides the performance of the professional participants separated into radiology residents and certified radiologists. For each measure, the statistics reported are from a 2 × 2 analysis of variance (ANOVA) with search display as a within-subjects factor (2D, 3D) and profession as a between-subjects factor (residents, certified radiologists). There were no differences between radiology residents and certified radiologists across key measures. There was a significant interaction effect between display type and profession for timeout rate; follow-up t-tests revealed no difference between the two groups on 2D trials (*t*(26) = 1.59, *p* = 0.12) or 3D trials (*t*(26) = 0.22, *p* = 0.82).

## Appendix 2

### Additional measures of interest from Experiment 1 and Experiment 2

**Table 3:** Summary of results for the other measures from professionals and non-professionals for Experiment 1

Other measures	Professionals		Non-professionals		Display type	Profession	Interaction
	2D	3D	2D	3D			
Timeout rate (%)	12.04	10.80	1.34	4.57	*p* = 0.54, η_p_^2^ = 0.01, BF_10_ = 0.25	*p* < 0.01, η_p_^2^ = 0.13, BF_10_ = 8.13	*p* = 0.17, η_p_^2^ = 0.03, BF_10_ = 0.68
Correct rejection rate (%)	73.53	91.79	72.69	97.31	*p* < 0.001, η_p_^2^ = 0.40, BF_10_ = 2.35 × 10^6^	*p* = 0.57, η_p_^2^ = 0.01, BF_10_ = 0.30	*p* = 0.37, η_p_^2^ = 0.02, BF_10_ = 0.45
Target-Present RT	19.36 s	26.94 s	21.70s	30.35 s	*p* < 0.001, η_p_^2^ = 0.35, BF_10_ = 3.83 × 10^3^	*p* = 0.22, η_p_^2^ = 0.03, BF_10_ = 0.47	*p* = 0.75, η_p_^2^ = 0.00, BF_10_ = 0.29

The statistics reported are based off a 2 × 2 ANOVA comparing search performance on 2D and segmented-3D trials across professionals and non-professionals from Experiment 1

*Timeout rate:* On average, the timeout rate was 11.42% for professionals (2D: 11.90%; 3D: 12.20%) and 2.95% for non-professionals (2D: 1.34%; 3D: 4.57%). There was a statistically significant main effect of profession (*F*(1,56) = 8.39, *p* = 0.005, η_p_^2^ = 0.13, BF_10_ = 8.13) with a higher timeout rate for professionals compared to non-professionals (see Appendix 2 Table 3). There was no main effect of display type (*F*(1,56) = 0.39, *p* = 0.54, η_p_^2^ = 0.01, BF_10_ = 0.25) and no significant interaction effect between display type and profession (*F*(1,56) = 1.95, *p* = 0.17, η_p_^2^ = 0.03, BF_10_ = 0.68).

*Correct rejection rate:* On average, the correct rejection rate was 79.60% for professionals (2D: 73.53%; 3D: 91.79%) and 85.00% for non-professionals (2D: 72.69%; 3D: 97.31%). One professional did not contribute to the final analysis as that individual timed out on all segmented-3D trial. There was no significant main effect of profession (*F*(1,55) = 0.33, *p* = 0.57, η_p_^2^ = 0.01, BF_10_ = 0.30), but there was a statistically significant main effect of display type (*F*(1,55) = 37.06, *p* < 0.001, η_p_^2^ = 0.40, BF_10_ = 2.35 × 10^6^) with a lower correct rejection rate for 2D search displays compared to segmented-3D displays (see Appendix 2 Table 3). There was no significant interaction effect between display type and profession (*F*(1,55) = 0.81, *p* = 0.37, η_p_^2^ = 0.02, BF_10_ = 0.45) (see Appendix 2 Table 4).

*Target-present response times:* On average, the target-present response time was 23.15 s for professionals (2D: 19.36 s; 3D: 26.95 s) and 26.03 s for non-professionals (2D: 21.70s; 3D: 30.35 s). Four professionals and six non-professionals did not contribute to the final analysis due to a combination of missing a target, making a false alarm, and/or having timed out on either all 2D or 3D trials. There was a statistically significant difference between display types (*F*(1,46) = 24.38, *p* < 0.001, η_p_^2^ = 0.35, BF_10_ = 3.83 × 10^3^) with a shorter target-present response time for 2D displays compared to 3D displays (see Table 1). There was no significant main effect of profession (*F*(1,46) = 1.54, *p* = 0.22, η_p_^2^ = 0.03, BF_10_ = 0.47) and no significant interaction between display type and profession (*F*(1,46) = 0.11, *p* = 0.75, η_p_^2^ = 0.002, BF_10_ = 0.29).

**Table 4:** Summary of results for the other measures for non-professionals from Experiment 2

Other measures	Experiment 2 Non-professionals		Display type comparison
	2D	3D	
Timeout rate (%)	0.43	1.58	*t*(28) = 2.12, *p* = 0.04, BF^10^ = 1.36
Correct rejection rate (%)	68.21	86.03	*t*(28) = 5.72, *p* < 0.001, BF^10^ = 5.08 × 10^3^
Target-present response time (s)	17.88	25.37	*t*(28) = 4.91, *p* < 0.001, BF^10^ = 6.73 × 10^2^

The statistics reported are based on t-tests comparing search performance on 2D and segmented-3D displays. There was a higher timeout rate, a higher correct rejection rate, and a longer average response time on target-present trials for segmented-3D displays compared to 2D displays

Non-professionals from Experiment 2 had an average timeout rate of 1.01% (2D: 0.43%; 3D: 1.58%). There was statistically significant difference between display types (*t*(28) = 2.12, *p* < 0.05, BF^10^ = 1.36) with fewer timeouts on 2D trials compared to 3D trials. They had an average correct rejection rate of 77.00% (2D: 68.21%; 3D: 86.03%). There was a statistically significant difference between display types (*t*(28) = 5.72, *p* < 0.001, BF^10^ = 5.08 × 10^3^) with a lower correct rejection rate on 2D trials compared to 3D trials. They had an average response time of 23.00s (2D: 17.88 s; 3D: 25.37 s) on target-present trials. There was a statistically significant difference between display types (*t*(28) = 4.91, *p* < 0.001, 6.73 × 10^2^) with a shorter target-present response time on 2D trials compared to 3D trials.

## Abbreviations

2D: Two-dimensional
3D: Three-dimensional

## Footnotes

1 We use the term “voxels” to indicate pixels that are in Z-dimension.

2 When search items appeared across multiple slices, they would become either lighter or darker from the previous slice depending on how many voxels of any particular item were averaged with the lighter background. This was done to give the impression of “depth” similar to how abnormalities can change in salience within a tomogram.

3 According to Wetzels et al., 2011, for a Bayes factor value in favor of the alternative hypothesis (in the current experiment that would be that there is a difference between searching in 2D displays and segmented-3D display), a value > 100 provides “decisive” evidence, 30–100 provides “very strong” evidence, 10–30 provides “strong” evidence, 3–10 provides “substantial” evidence, and 1–3 provides “anecdotal” evidence in favor of the alternative hypothesis. Likewise, for a Bayes factor value in favor of the null hypothesis (i.e. that there is no difference between searching in 2D displays and segmented-3D display), a value < 1/100 provides “decisive” evidence, 1/100–1/30 provides “very strong” evidence, 1/30–1/10 provides “strong” evidence, 1/10–1/3 provides “substantial” evidence, and 1/3–1 provides “anecdotal” evidence null hypothesis.

4 A’ and B” are measures used for non-parametric data (Snodgrass and Corwin, 1988). These measures were used because more than half the participants did not false alarm in the target absent, segmented-3D condition. A’ indicates how easy it was to identify an item as a target and the B” indicates how willing an observer was to say a target was present at the cost of making a false alarm. An A’ measure of 0.5 signifies chance performance and a B” measure of 0 signifies no bias.
